# Relating inter-individual differences in metacognitive performance on different perceptual tasks

**DOI:** 10.1016/j.concog.2010.12.011

**Published:** 2011-12

**Authors:** Chen Song, Ryota Kanai, Stephen M. Fleming, Rimona S. Weil, D. Samuel Schwarzkopf, Geraint Rees

**Affiliations:** a UCL Institute of Cognitive Neuroscience, University College London, 17 Queen Square, London WC1N 3AR, UK; b Wellcome Trust Centre for Neuroimaging, University College London, 12 Queen Square, London WC1N 3BG, UK

**Keywords:** Metacognition, Consciousness, Perception, Inter-individual difference, Signal detection theory

## Abstract

Human behavior depends on the ability to effectively introspect about our performance. For simple perceptual decisions, this introspective or metacognitive ability varies substantially across individuals and is correlated with the structure of focal areas in prefrontal cortex. This raises the possibility that the ability to introspect about different perceptual decisions might be mediated by a common cognitive process. To test this hypothesis, we examined whether inter-individual differences in metacognitive ability were correlated across two different perceptual tasks where individuals made judgments about different and unrelated visual stimulus properties. We found that inter-individual differences were strongly correlated between the two tasks for metacognitive ability but not objective performance. Such stability of an individual’s metacognitive ability across different perceptual tasks indicates a general mechanism supporting metacognition independent of the specific task.

## 1 Introduction

Virtually everyone, in their everyday lives, regularly makes decisions upon their knowledge of the outside world and the confidence they have in their knowledge. For example, deciding whether or not to operate on a brain tumor depends on the confidence a neurosurgeon has in their surgical competency. Similarly, a student facing the choice of university major will base their decision upon a self-evaluation of their strengths and weakness. This knowledge about one’s own cognition is known as metacognitive knowledge (Flavell, 1979; Metcalfe & Shimamura, 1994). Metacognitive ability is considered as an important index of intelligence (Borkowski, Carr, & Pressely, 1987) and consciousness (Persaud, McLeod, & Cowey, 2007), and a capacity more advanced in humans than animals (Smith, 2009; Terrace & Son, 2009).

Metacognitive ability can be expressed as the mapping between objective performance, such as a perceptual judgment on the duration of a flash, and subjective confidence in the accuracy of such an objective judgment (Kunimoto, Miller, & Pashler, 2001; Galvin, Podd, Drga, & Whitmore, 2003; Lau & Passingham, 2006). Just as the ability of making correct objective judgments varies substantially among individuals (Halpern, Andrews, & Purves, 1999; Kirchhoff & Buckner, 2006), the metacognitive ability of determining whether these judgments are correct also shows individual differences. While some people can give an accurate evaluation on their own performance or knowledge, others consistently fail to do so. A recent study showed a striking link between such metacognitive differences and the anatomical structure of rostral prefrontal cortex (Fleming, Weil, Nagy, Dolan, & Rees, 2010). This study suggests a focal neuroanatomical basis for metacognitive ability (at least in the perceptual task that was studied) but did not address whether the inter-individual variability in metacognitive ability observed was related specifically to the perceptual task on which the participants evaluated their performance, or might generalize to other tasks.

In general, an individual’s performance depends on the perceptual task involved, even for apparently related perceptual tasks. For example, individuals who perform well at orientation discrimination do not necessarily perform well on motion discrimination (Halpern et al., 1999). Whether similar differences exist for metacognitive knowledge about these tasks is not clear. Here, we therefore compared the metacognitive ability of a group of individuals independently evaluating their performance on two different perceptual tasks. To anticipate our findings, we found that while individuals’ visual performance differed substantially across the two perceptual tasks (i.e., individuals doing well in one task may not do well in the other task), metacognitive ability was instead consistent across the different tasks (i.e., individuals with high metacognitive ability in one task had similarly high metacognitive ability in the other task). In contrast to the traditional framework suggesting the involvement of task-specific brain mechanisms (Nelson & Narens, 1990), our study indicates that metacognitive ability may be mediated, at least for visual perceptual tasks, by a single brain mechanism.

## 2 Material and methods

**Fig. 1:**
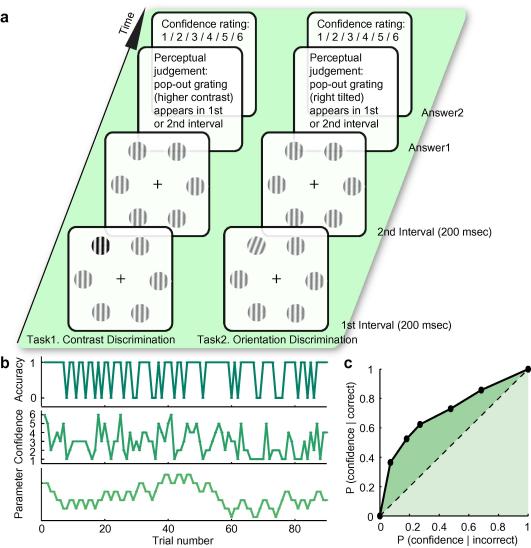
Schematic depiction of experiment paradigm. (a) Participants made a two-interval forced choice judgment on which temporal interval contained the grating that popped out in contrast (task one) or orientation (task two). Following their objective perceptual judgment, participants also provided a rating of confidence in their visual performance. (b) To induce uncertainty about the visual judgments, the parameter (contrast in task one, orientation in task two) of the pop-out grating was varied in the 2-up-1-down staircase fashion to keep each participant’s performance near threshold. The participants’ visual performance was evaluated by the average parameter of the pop-out grating, i.e., the discrimination threshold. (c) Metacognitive ability was quantified by the interrelationship between confidence ratings and the accuracy of visual judgments using the type II receiver operating characteristic (ROC) curve, which characterized the probability of being correct for a given level of confidence.

We studied the metacognitive abilities of individuals performing two different visual tasks – a contrast discrimination task and an orientation discrimination task (Fig. 1a). On a trial-by-trial basis, participants made objective perceptual judgments on the contrast or the orientation of the visual stimuli, and assessed their confidence in the accuracy of perceptual judgments. The interrelationship between the perceptual judgment and the confidence rating reflects their metacognitive ability.

### 2.1 Participants

Eighteen healthy volunteers (10 females, 8 males, aged 19–33) with normal or corrected-to-normal vision participated in this study. All gave written informed consent, and the study was approved by the local ethics committee.

### 2.2 Stimuli

In both tasks, visual stimuli comprised six vertical gratings (2.8 visual degrees in diameter, spatial frequency of 2.2 cycles per visual degree, contrast of 20%) arranged in a circular fashion (eccentricity of 6.9 visual degrees) around a central fixation cross. The stimuli were presented on a calibrated CRT monitor (size 22″, spatial resolution of 1024 × 768 pixels, refresh rate of 100 Hz, viewing distance of 67 cm), and the experiment was conducted in a darkened room with the monitor providing the only significant source of light. The stimuli and the experiment were programmed in MATLAB (Mathworks Inc., Natick, MA, USA) using Psychtoolbox (Brainard, 1997).

### 2.3 Procedure

On each trial, the visual stimuli were presented twice with each presentation lasting 200 ms and the inter-stimulus interval lasting 500 ms. In one of the two presentation intervals, the six gratings were exactly the same, but in the other interval, one of the six gratings differed from the rest by a higher contrast (task one) or slightly different orientation (task two). The temporal interval and spatial position of the pop-out grating varied randomly between trials.

Participants made an unspeeded two-interval forced choice judgment as to which interval contained the pop-out grating. In addition to making the objective visual judgments, participants provided ratings of confidence in their perceptual decisions on a scale of 1 (low confidence) to 6 (high confidence) (Fleming et al., 2010). The difficulty of the visual judgments, i.e., the parameter (contrast in task one, orientation in task two) of the pop-out grating, was varied in a 2-up-1-down staircase fashion (Levitt, 1971) to keep each participant in the near threshold region (such that performance converged on 70.7% correct) (Fig. 1b). Two consecutive correct visual judgments led to the parameter of the pop-out grating in the next trial being one step lower than in the previous trials, whereas one incorrect visual judgment led to an increase in the parameter of the pop-out grating. The step size was 2% (contrast, task one) or 0.5 degree (orientation, task two). Participants were instructed to use the whole confidence scale and to bear in mind that the scale represented relative confidence, since given the difficult nature of the visual tasks, they would rarely be completely certain that they made a correct visual judgment.

For each task, participants completed 540 trials (split into 6 blocks of 90 trials). The starting parameter of the pop-out grating in the first block was the discrimination threshold measured through a preliminary experiment that involved visual judgments without confidence rating. In the following blocks, the starting parameter was the discrimination threshold in the last block. In order to control practice effects that may unevenly influence individual performance in the first versus second task, the experiment sequence was the same for all participants.

### 2.4 Data analysis

Visual performance was evaluated by calculating the contrast (task one) and orientation (task two) discrimination thresholds, at which the accuracy of visual judgements converged to 70.7% as a result of the 2-up-1-down staircase procedure (Levitt, 1971). The discrimination threshold was calculated as the average contrast or orientation of the pop-out grating.

Metacognitive ability was quantified by the interrelationship of the confidence rating and the accuracy of visual judgments using the type II receiver operating characteristic (ROC) curve (Fig. 1c) (Galvin et al., 2003; Kornbrot, 2006; Fleming et al., 2010). The type II ROC curve characterized the probability of being correct for a given level of confidence. To construct the ROC curve, *p* (confidence = *i* | correct) and *p* (confidence = *i* | incorrect) were calculated for all *i*, and were then transformed into cumulative probabilities and plotted against each other. The area underlying the ROC curve quantified the metacognitive ability. This area was calculated by the sum of the area between the ROC curve and the diagonal (dark green^1^ area in Fig. 1c) and the area of the half-square triangle (light green area in Fig. 1c): $A_{\mathrm{ROC}}=0.25\sum_{i=1}^{6}\left[{\left(Y_{i+1}-X_{i}\right)}^{2}-{\left(Y_{i}-X_{i+1}\right)}^{2}\right]+0.5$ (Kornbrot, 2006). The mean confidence rating was also calculated for each participant in each task. It reflects the bias an individual has towards using higher or lower confidence ratings. The mean confidence rating correlates very well with the confidence bias reflected by the ROC curve: $B_{\mathrm{ROC}}=\ln\left((\sum_{i=1}^{3}\left[{\left(Y_{i+1}-X_{i}\right)}^{2}-{\left(Y_{i}-X_{i+1}\right)}^{2}\right]/\sum_{i=4}^{6}\left[{\left(Y_{i+1}-X_{i}\right)}^{2}-{\left(Y_{i}-X_{i+1}\right)}^{2}\right]\right)$ (*r = *0.94, *p *< 10^−18^). The ROC model provided a good fit to our data (for the linear regression between *z*(*Y*) and *z*(*X*), mean *R*^2^ = 0.985 ± 0.003, mean slope = 0.809 ± 0.024).

## 3 Results

For both tasks (contrast discrimination and orientation discrimination), we found considerable inter-individual variability in visual performance (contrast discrimination threshold from 7% to 15%, orientation discrimination threshold from 1.7 degrees to 3.4 degrees). We also found similarly large variability across participants in their metacognitive ability (*A_ROC_* = 0.62–0.80), confidence level (average confidence rating = 1.6–4.5). Thus, our tasks produced a variable range of objective performance and metacognitive ability that were neither at ceiling nor floor.

**Fig. 2:**
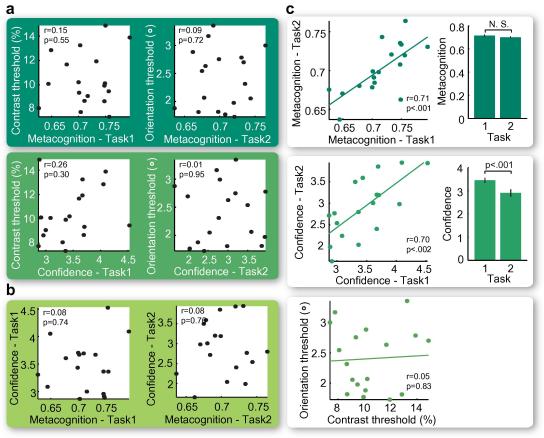
Experiment results. (a) For both tasks, there existed no correlation between visual performance and metacognitive ability or mean confidence level. (b) Metacognitive ability and confidence level were not correlated. (c) Though participants’ visual performance was not correlated across two tasks, correlations were observed between task-specific metacognitive abilities, and between task-specific mean confidence levels. Moreover, while metacognitive ability did not show significant difference between two tasks, mean confidence level was much lower in task two (orientation discrimination task) than task one (contrast discrimination task).

Across participants, neither metacognitive ability nor confidence level correlated with objective performance (correlation between metacognitive ability and visual performance, task one: *r *= 0.15, *p *= 0.55, task two: *r = *0.09, *p *= 0.72; correlation between confidence level and visual performance, task one: *r *= 0.26, *p *= 0.30, task two: *r = *0.01, *p *= 0.95; Fig. 2a), establishing that the measurement of an individual’s metacognitive ability was not confounded by the stimulus parameters in the visual tasks.

When we compared individuals’ performance across the two tasks, we found no correlation in their objective visual performance (*r *= 0.05, *p *= 0.83, Fig. 2c). However, metacognitive ability showed a highly significant correlation between the two different tasks (*r *= 0.71, *p *< 0.001, Fig. 2c), as did confidence level (*r *= 0.70, *p *< 0.002, Fig. 2c). Interestingly, the mean metacognitive ability across participants did not show any significant difference between the two tasks (*t*(17) = 1.8, *p *= 0.094, Fig. 2c), whereas the average confidence level was much lower in the orientation discrimination task compared with the contrast discrimination task (*t*(17) = 4.7, *p *< 0.001, Fig. 2c). This indicates that the confidence level consists of a task-independent component which reflects the general confidence one has (i.e., individuals gave high/low confidence ratings in one task tended to gave high/low ratings in the other task), as well as a task-dependent component which reflects the confidence one has towards a specific task and likely correlates with one’s feeling of task difficulty. Therefore, while across participants, objective performance was dependent on the specific task (contrast or orientation discrimination), the metacognitive ability exhibited by our participants was task-independent; and their confidence levels reflected both task-independent self-confidence as well as the evaluation of task difficulty.

We also found that metacognitive ability and confidence level were not correlated (task one: *r *= 0.08, *p *= 0.74; task two: *r *= 0.08, *p *= 0.76; Fig. 2b). Specifically, individuals with higher confidence were not better at evaluating their performance and vice versa. This further supports a signal detection theoretic approach where metacognitive ability (sensitivity) and the confidence level (bias) are considered as two independent properties (Galvin et al., 2003).

## 4 Discussion

An interesting question in metacognition is its relationship with more fundamental aspects of cognition such as visual perception. By definition, metacognition is cognition about cognition (Metcalfe & Shimamura, 1994), implying a mutual relationship between metacognition and cognition. Indeed, a traditional view holds that metacognition represents the dynamical interplay between a cognition-level and a meta-level (Nelson & Narens, 1990). The cognition-level provides the meta-level with accessible information for introspection and updates the meta-level’s model of the cognition-level, whereas information flowing back from the meta-level implements control over the cognition-level such as terminating a cognition-level behavior. This framework thus predicts that introspective or metacognitive ability is mediated by several brain processes, including those responsible for different perceptual decisions, and that metacognitive performance might differ when comparing different perceptual or cognitive tasks.

However, we found that an individual’s metacognitive ability measured in one visual task could predict their metacognitive ability in a second visual task, independent of the variability in their objective performance on perceptual decisions. This result suggests that metacognitive ability, at least for the tasks studied, shares a common cognitive process independent of the perceptual task. It is also consistent with observations that the ability to introspect about perceptual decisions is correlated with the anatomical microstructure of a single cortical location dissociated from those concerned with visual perception such as the sensory cortices (Fleming et al., 2010). One limitation of our study is the relative similarity between two perceptual tasks. Participants’ objective performances were not correlated across two tasks, indicating that the correlation we observed in metacognitive ability is unlikely to result from common mechanisms for perceptual decision making. Nevertheless, the two perceptual tasks both involve visual processing and share similar paradigms. It would be of interest for future studies to now address whether metacognitive ability shows a similar correlation between tasks involving different perceptual modalities, such as vision and audition.

Parallel to our work, a recent experiment reported a dissociation between metacognitive and cognitive performance (Rounis, Maniscalco, Rothwell, Passingham, & Lau, 2010). Specifically, altering metacognition either through visual masking or transcranial magnetic stimulation (TMS) of dorsal-lateral prefrontal cortex does not seem to affect visual performance per se (Lau & Passingham, 2006; Rounis et al., 2010). These studies showed the constancy in perceptual performance under shifts in metacognitive performance. However, empirical support for the constancy of metacognition under changes in cognitive task has been lacking, and our study provides the first evidence for this. Together these lines of new evidence suggest the independence of metacognitive awareness from primary cognition (Lau, 2008).

The dissociation of metacognition from task-specific systems has direct implications for the conceptual framework of consciousness (Crick & Koch, 2003; Ramsoy & Overgaard, 2004; Tononi, 2008; Lau, 2008). Metacognitive ability has been widely used as an assessment of perceptual awareness (Poppel, Held, & Frost, 1973; Trevethan, Sahraie, & Weiskrantz, 2007; Persaud et al., 2007; Koch & Preuschoff, 2007; Kanai, Walsh, & Tseng, 2010). In the absence of awareness, such as in the case of blindsight, patients often claim no subjective experience of seeing despite their above-chance accuracy in visual discrimination (Poppel et al., 1973; Trevethan et al., 2007; Persaud et al., 2007). These observations have led to proposals that consciousness is not reflected in the perceptual processing per se (e.g., high accuracy in visual discrimination) but rather in the introspective awareness of perceptual processing (i.e. metacognitive ability) (Ramsoy & Overgaard, 2004; Lau, 2008). Thus, a key neural component for introspective awareness may be the prefrontal cortical regions associated with metacognitive ability (Del Cul, Dehaene, Reyes, Bravo, & Slachevsky, 2009; Fleming et al., 2010; Rounis et al., 2010).

Since metacognitive ability is an individual attribute dissociable from performance on perceptual tasks, our study further suggests the use of metacognitive ability as a method of quantifying higher-order aspects of consciousness (Ramsoy & Overgaard, 2004; Lau, 2008). Intuitively, confidence in perceptual performance requires monitoring not only the perceptual stimulation, but also one’s response to the stimulation. Thus, an individual’s metacognitive ability may be partially separable from perceptual processing (Sandberg, Timmermans, Overggard, & Cleeremans, 2010), and may additionally rely on the efficacy of response monitoring mechanisms (Yeung, Botvinick, & Cohen, 2004). The relationship between these constructs remains to be determined by future studies.

## 5 Conclusions

In conclusion, we found that individuals’ metacognitive knowledge of perceptual performance remained stable across two different visual tasks, even though the individual objective performance changed substantially between different tasks. This indicates that the higher-order metacognitive ability is likely to be mediated by a general mechanism independent of the first-order cognitive task.

## Footnotes

1 For interpretation of color in Figs. 1 and 2, the reader is referred to the web version of this article.
